# Enhanced NH_3_ Sensing Performance of Mo Cluster-MoS_2_ Nanocomposite Thin Films via the Sulfurization of Mo_6_ Cluster Iodides Precursor

**DOI:** 10.3390/nano13030478

**Published:** 2023-01-24

**Authors:** Meiqi Zhang, Fabien Grasset, Yuji Masubuchi, Toshihiro Shimada, Thi Kim Ngan Nguyen, Noée Dumait, Adèle Renaud, Stéphane Cordier, David Berthebaud, Jean-François Halet, Tetsuo Uchikoshi

**Affiliations:** 1 Graduate School of Chemical Sciences and Engineering, Hokkaido University, Kita 13 Nishi 8, Kita-ku, Sapporo 060-8628, Japan; 2 Research Center for Functional Materials, National Institute for Materials Science, 1-2-1 Sengen, Tsukuba 305-0047, Japan; 3 CNRS–Saint-Gobain–NIMS, IRL3629, Laboratory for Innovative Key Materials and Structures (LINK), National Institute for Materials Science, 1-1 Namiki, Tsukuba 305-0044, Japan; 4 Univ Rennes, CNRS, Institut des Sciences Chimiques de Rennes (ISCR)–UMR 6226, F-35000 Rennes, France; 5 Division of Applied Chemistry, Faculty of Engineering, Hokkaido University, Kita 13 Nishi 8, Kita-ku, Sapporo 060-8628, Japan; 6 International Center for Young Scientists, ICYS-SENGEN, Global Networking Division, National Institute for Materials Science, 1-2-1 Sengen, Tsukuba 305-0047, Japan

**Keywords:** molybdenum octahedral cluster, molybdenum disulfide, sulfurization process, gas sensor

## Abstract

The high-performance defect-rich MoS_2_ dominated by sulfur vacancies as well as Mo-rich environments have been extensively studied in many fields, such as nitrogen reduction reactions, hydrogen evolution reactions, as well as sensing devices for NH_3_, which are attributed to the under-coordinated Mo atoms playing a significant role as catalytic sites in the defect area. In this study, the Mo cluster-MoS_2_ composite was creatively synthesized through a one-step sulfurization process via H_2_/H_2_S gas flow. The Mo_6_ cluster iodides (MIs) coated on the fluorine-doped tin oxide (FTO) glass substrate via the electrophoretic deposition method (i.e., MI@FTO) were used as a precursor to form a thin-film nanocomposite. Investigations into the structure, reaction mechanism, and NH_3_ gas sensing performance were carried out in detail. The results indicated that during the gas flowing, the decomposed Mo_6_ cluster iodides played the role of template and precursor, forming complicated Mo cluster compounds and eventually producing MoS_2_. These Mo cluster-MoS_2_ thin-film nanocomposites were fabricated and applied as gas sensors for the first time. It turns out that after the sulfurization process, the response of MI@FTO for NH_3_ gas increased three times while showing conversion from p-type to n-type semiconductor, which enhances their possibilities for future device applications.

## 1. Introduction

Graphene-like molybdenum disulfide (MoS_2_), with its well-known two-dimensional (2D) layered “sandwich” structure, has attracted significant attention over the years due to its unique electronic, optical, and chemical properties [1,2,3,4], which can be exploited in transistors [5,6], solar cells [7,8], catalysts [9,10,11], and sensors [12,13,14]. In this “sandwich” structure, one layer of molybdenum atoms is sandwiched between two layers of sulfur atoms. In the domain of catalysis, such MoS_2_ three-layer structures have been widely investigated for their catalytic activity, which is strongly dependent on the edge sites [15]. To enhance the catalytic performance, researchers studied the synthesis of defect-rich MoS_2_, in which a sulfur vacancy that created the edge sites plays an important role [16,17,18]. Indeed, at the sulfur vacancy sites, the surrounding under-coordinated Mo atoms introduce gap states that facilitate N_2_ absorption or hydrogen bonding, which improves the performance of the nitrogen reduction reaction (NRR) [19], hydrogen evolution reaction (HER) [20,21], as well as sensing [22,23,24]. 

With the development of defect engineering, researchers have devoted themselves to the synthesis of S vacancies defect-rich MoS_2_ by using the hydrothermal route [25,26] and the chemical vapor deposition (CVD) method [27], which can control the sulfur-molybdenum ratio during the reaction to create defects. Alternatively, 2D-MoS_2_ decorated with Mo clusters has started to attract some attention as a catalyst for the generation of acetylene via the CO hydrogenation reaction [28]. A higher electrocatalytic NRR activity has been theoretically proposed using Mo_x_ clusters (x = 1~11) supported on MoS_2_ [29]. Moreover, Jin et al. demonstrated the in situ fabrication of Mo_6_S_6_-nanowire-terminated edges in the monolayer MoS_2_ [30] and developed a mass transport method for the formation of Mo chains induced by Mo clusters along the sulfur vacancy lines, aimed at tuning the defects and, thus, the materials’ structure–property relationship in a well-controlled manner [31]. However, only a few studies have been conducted to generate MoS_2_ by sulfuring chalcogenide clusters, such as Mo_6_S_2_I_8_, as precursors [32,33]. 

In this study, we creatively pioneered the efficient synthesis route of Mo cluster-MoS_2_ thin-film nanocomposites by a sulfurization process using Mo_6_ cluster iodides as the precursors. Mo_6_ cluster units are inorganic molecules containing six Mo atoms forming an octahedral skeleton that are connected by direct metal–metal bonds and are bonded with organic or inorganic ligands to stabilize the metal core. The interest in Mo_6_ clusters-based compounds started with the discovery of the superconductivity in the Chevrel phases [34]. In recent decades, the optical properties and the chemical activity of Mo_6_ clusters derived from their unique electronic structure [35,36,37] have been investigated and developed in many fields [38], such as luminescent [39,40,41], energy [42], and catalysis applications [43]. Among them, Nguyen et al. successfully developed a general process to coat metal-cluster-unit-based thin films, for example, Mo_6_ cluster iodides (Cs_2_Mo_6_I_14_, CMI; more specifically, Cs^+^_2_[{Mo_6_I^i^_8_}I^a^_6_]^2-^, where i and a represent the inner and apical iodide ligands, respectively, that surround the Mo_6_ octahedra) on a fluorine-doped tin oxide (SnO_2_:F, FTO) glass substrate by the electrophoretic deposition (EPD) method [44]. This EPD method maintained the optoelectronic properties of the initial CMI raw powder, which will significantly promote the fabrication of Mo_6_-cluster-based optoelectronic devices in the future. Moreover, Renaud et al. recently proved that the Mo_6_ cluster iodides are a new class of ambipolar materials [45], and the {Mo_6_I^i^_8_}-based thin films (noted MI@FTO) fabricated via the EPD method have shown a significant application potential as a photoabsorber in an all-solid solar cell in the future. Moreover, Guy et al. used various Mo_6_ clusters powders (Cs_2_Mo_6_CI_14_, (TBA)_2_Mo_6_Br_14_, etc.) as precursors to synthesize Mo_x_N_y_ nanomaterials with different compositions (Mo_2_N and Mo_5_N_6_) [46]. In contrast to the conventional synthesis using MoO_3_ as a precursor, these Mo_x_N_y_ products were prepared at lower temperatures and showed significant catalytic performances for the water–gas shift reaction. 

In a previous study, we successfully produced MoS_2_ from Mo_6_Br_12_ powder as a precursor via two sulfurization processes [47]. Using the gas flow method, the sulfurization reaction occurred at 250 °C, but the product was a micron-sized amorphous MoS_2_ powder. This reaction process still needs to be developed and the reaction mechanism better understood. Therefore, in this study, we decided to use this gas flow method combined with the EPD technology to establish a one-step sulfurization method at low temperature to fabricate a Mo cluster–MoS_2_ thin film nanocomposite by using pre-prepared MI@FTO coatings as the precursor. The morphology and the reaction mechanism were investigated in detail. For future device applications, the obtained thin-film samples were carefully evaluated for their NH_3_ gas sensing performance. The main results are reported and discussed.

## 2. Materials and Methods

### 2.1. Chemicals

The synthesis path of the Cs_2_Mo_6_I_14_ initial powders via the solid-state method [48,49] and the MI@FTO films fabricated by using the EPD method [50] have been well established in our previous studies. 

Briefly, Cs_2_Mo_6_I_14_ was synthesized by reacting CsI and MoI_2_ mixed and heated at 700 ℃ for 4 days in a silica tube sealed under vacuum. For the EPD process, the Cs_2_Mo_6_I_14_ raw powder was dissolved in acetone (reagent-grade chemicals, 99.5%) at the concentration of 5 g per liter. The solution was then filtered using a 0.2 μm filter to remove any insoluble impurities. At the same time, the FTO glass substrate (Kenis Corp., Osaka, Japan) was cut into a 25 mm × 10 mm piece and washed in detergent and acetone for 30 min in an ultrasonic bath. Finally, the FTO substrate was used as the anode and immersed in a Cs_2_Mo_6_I_14_ cluster solution. The coating process was performed at 10 V for 5 min. Cs^+^ cations and [{Mo_6_I^i^_8_}X^a^_6_]^2−^ (X = I, OH) were deposited on the cathode and the FTO glass anode, respectively [45]. It should be noted that during the EPD coating process, the [{Mo_6_I^i^_8_}I^a^_6_]^2−^ cluster molecules will lose two iodide apical ligands, which were replaced by water ligands, as confirmed by the XPS result in a previously published report [45]. Accordingly, the final composition of the MI@FTO film in this study is described as [{Mo_6_I^i^_8_}I^a^_4_(H_2_O)^a^_2_]·xH_2_O. The MI@FTO thin films deposited on the FTO substrate were used for the sulfurization process. 

### 2.2. Sulfurization Process

**Figure 1:**
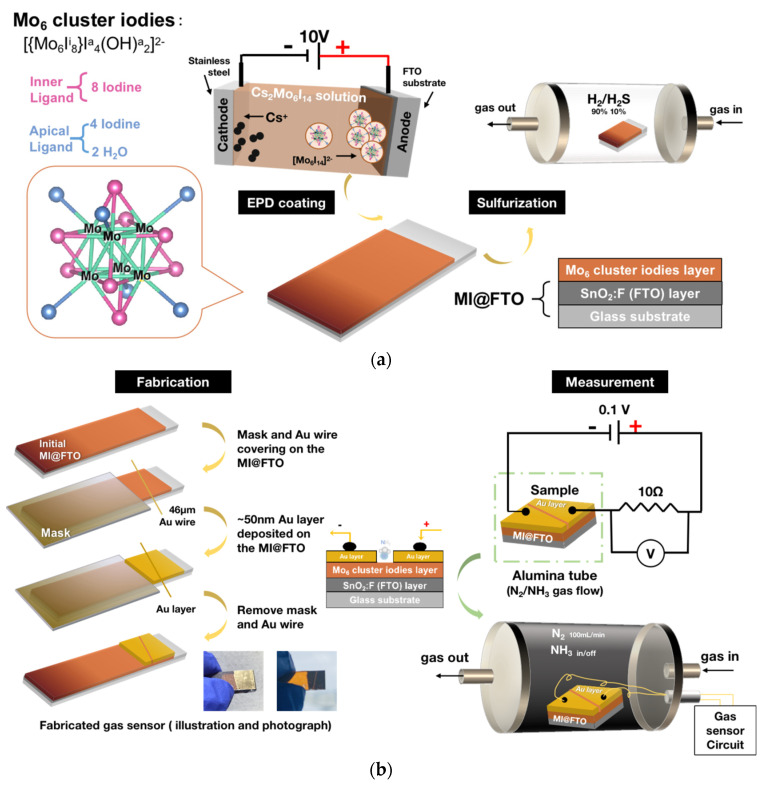
(**a**) Illustration of MI@FTO prepared via EPD and sulfurization processes. (**b**) Illustration of the gas sensor fabrication and measurement.

All the sulfurization reactions were carried out in a tubular furnace with a flowing H_2_/H_2_S (90%/10%) mixture gas (99.9%, Air Liquid). The MI@FTO sample was initially placed in an aluminum boat, then into the furnace. The reaction chamber was purged with N_2_ gas for 30 min before the start of the reaction to ensure removal of the air, and the furnace was always at 1 atmospheric pressure. The H_2_/H_2_S gas was then introduced at the rate of 15 mL/min in the temperature range of 250–450 °C, and the main reaction time was 1 h for all the MI@FTO films with the increasing rate of 5 degrees per minute. The H_2_S acted as the sulfur source for the sulfurization reaction and the H_2_ acted as the reducing atmosphere to protect the cluster film from oxidation. An illustration of the EPD and the sulfurization process for the MI@FTO is shown in Figure 1a. According to the reaction temperature, the final products were denoted as MI-250, MI-300, etc. The reaction time was labeled for the specific sample, which is the extended reaction times, e.g., MI-350-3h.

### 2.3. Fabrication and Measurement of Gas Sensor

The fabrication and measurement of the MI@FTO gas sensor are illustrated in Figure 1b. This measurement was conducted by a chemiresistive gas sensor, which consisted of two electrodes connected to the sensing material. The change in resistance or current of the sensor device was measured in order to analyze the concentration of the target gas.

First, a 46 μm Au wire covered the MI@FTO surface, and the covered area was used for the performance measurement. An approximately 50 nm Au layer was deposited on the exposed sample surface by a vacuum evaporator (VG Microtech E6700S, Newhaven, UK) to improve the surface conductivity. A Cu wire was connected from the Au-deposited area to the ampere-meter (Graphtec midi Logger GL240, Graphtec Corporation, Yokohama, Japan) to monitor and record the real-time current. The MI@FTO was placed in an alumina tube with flowing NH_3_ gas (99.9%, Sumitomo Seika Chemicals, Tokyo, Japan) for the measurements.

### 2.4. Characterization

The structure and morphology of the thin-film samples, before and after sulfurization, were investigated by the grazing-incidence X-ray diffraction technique (GIXRD, SmartLab, Rigaku Corp., Tokyo, Japan) at room temperature using Cu Kα radiation (λ = 1.5406 Å) at 50 mA and 40 kV power, and the incident beam was set at fixed critical w angles (0.2° ≤ w ≤ 0.8°). The layered structures were analyzed by micro-Raman spectroscopy (HORIBA T64000, Kyoto, Japan) using a 515 nm laser source. The surface morphology and the elemental composition mapping of the thin-film sample were analyzed by field emission scanning electron microscopy (FE-SEM, Hitachi SU4800, Tokyo, Japan) at 10–15 kV coupled with an energy-dispersive X-ray (EDX) analysis device, and high-resolution observations of the powder scratched from the thin-film sample were performed by a high-resolution transmission electron microscope (HRTEM, JEOL 2100F, Tokyo, Japan) equipped with an EDX analysis device. The electron binding energy spectra of the thin-film samples were measured by X-ray photoelectron spectroscopy (XPS, ULVAC Quantum-2000, Chigasaki, Japan) using Al Kα radiation at 15 kV and 50 W and a take-off angle of 45°. All the binding energies were calibrated versus the C 1s peak of the adventitious carbon at 285 eV.

## 3. Results and Discussion

### 3.1. Structure and Morphology

**Figure 2:**
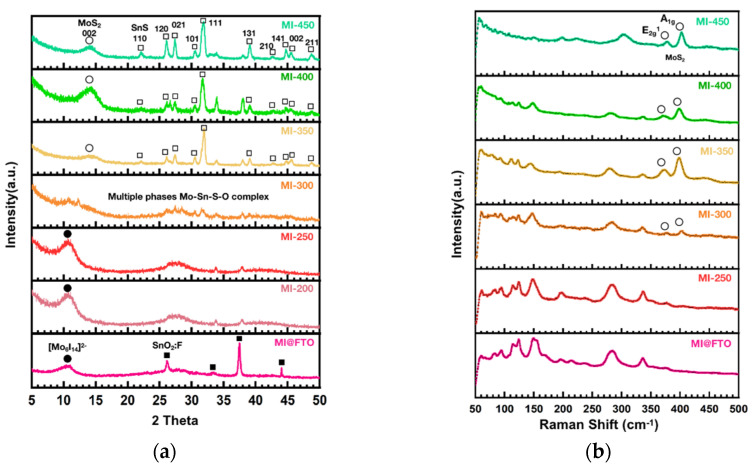
(**a**) GIXRD pattern of MI@FTO and sulfurized samples at different temperatures; (**b**) Raman spectra of MI@FTO and sulfurized samples under 515 nm excitation.

The GIXRD results in Figure 2a show the patterns of the initial MI@FTO and sulfurized samples. The wide peak observed around 9–12 degrees and the sharp crystal peaks at the higher 2θ angle in the initial MI@FTO pattern can be identified as the amorphous Mo_6_ cluster iodide film and SnO_2_:F from the FTO substrate, respectively, which is consistent with our previous studies [45,51]. MoS_2_ and SnS are found as the final products during this sulfurization process (see MI-450 pattern in Figure 2a). Indeed, MoS_2_ is the final product of the sulfurization reaction starting from the MI@FTO thin-film precursor occurring from 250 °C, and this result is consistent with the powdered Mo_6_Br_12_ cluster precursor [47]. The wide peak initially measured around 9–12 degrees, due to the Mo_6_ cluster iodides, has shifted to 14 degrees upon increasing the temperature. This indicates that the Mo_6_ cluster iodide has entirely disappeared in favor of MoS_2_. The presence of SnS crystals over 350 °C is unexpected. The SnO_2_ layer between the Mo_6_ cluster iodides and the SiO_2_ glass layer plays the role of the Sn source leading to SnS crystals at the end of the process. The pattern of MI-300 shows complex peaks of multiple phases. Considering that SnO_2_ may have been involved in the reaction, the product might contain the Mo_x_S_y_ complex [52], the Sn_x_S_y_ complexes [53], and also the Sn-Mo cluster complexes [54]. Additionally, the involvement of an O source derived from SnO_2_ may also have contributed to the formation of metal oxysulfide complexes [55]. In general, the FTO substrates are used as substrates for the synthesis of tin sulfides using the CVD method because the SnO_2_ surface is suitable for the growth of tin sulfides and does not participate in the reaction at such temperatures [56,57]. This fact implies that the excellent catalytic properties of the Mo_6_ cluster iodides promote the SnO_2_ layer in order to participate in the sulfurization reaction [57].

In contrast to the well-grown SnS crystal, the final product of the reaction, only one wide diffraction pattern present in the XRD patterns was used to confirm the formation of MoS_2_. In the Raman spectrum (see Figure 2b), the MoS_2_ layered structure peaks are observed around 375 cm^−1^ and 400 cm^−1^, which represent the in-plane vibration (E_2g_^1^) and out-of-plane vibration (A_1g_), respectively, beyond 300 °C. It is indicated that the Mo_6_ cluster iodides, used as a precursor, are gradually sulfurized into the layered MoS_2_ structure, which is consistent with the wide peak around 14 degrees corresponding to the MoS_2_ (002) surface in the XRD pattern.

**Figure 3:**
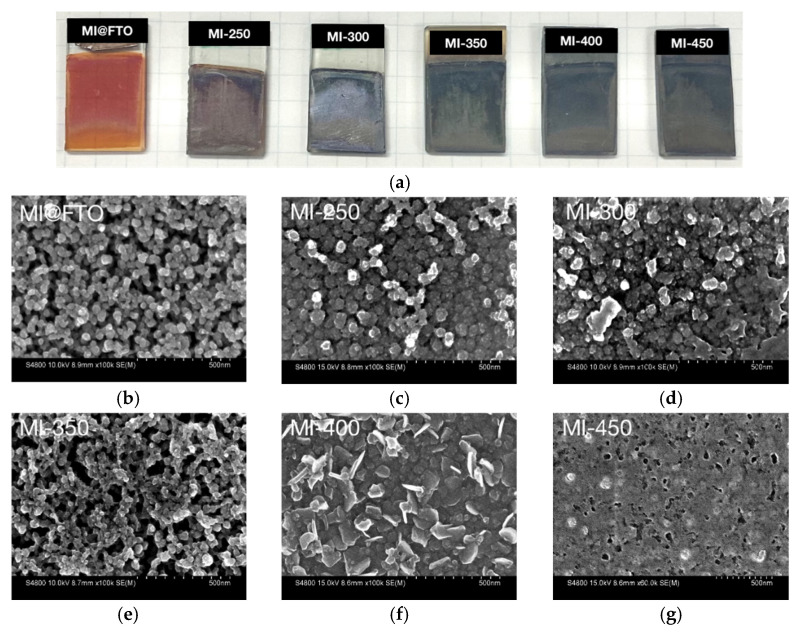
Pictures and FE-SEM observation with the scale bar at 500 nm of MI@FTO and sulfurized samples: (**a**) photographs of samples on a squared paper (5 mm × 5 mm); (**b**) initial MI@FTO; (**c**) MI-250; (**d**) MI-300; (**e**) MI-350; (**f**) MI-400; (**g**) MI-450.

Figure 3a shows pictures of the initial MI@FTO and sulfurized samples at different temperatures. The initial MI@FTO film is a translucent orange film. During the sulfurization process, the color of the MI@FTO films undergo a change from orange to dark brown (MI-250) to dark gray with a metallic luster (from MI-300 to MI-450), which indicates that the sulfurization reaction occurs beyond 250 °C. The dark gray color of the metallic luster sample must also correspond to the MoS_2_ and SnS phases. The surface morphology of the initial MI@FTO observed by FE-SEM and illustrated in Figure 3b shows isolated [{Mo_6_I^i^_8_}X^a^_6_]^2−^ (X = I, OH) nanoparticles with diameters around 20–50 nm up to 300 °C. As the temperature increases to 350 °C, the nanoparticles are sintered into a continuous porous structure. 

**Figure 4:**
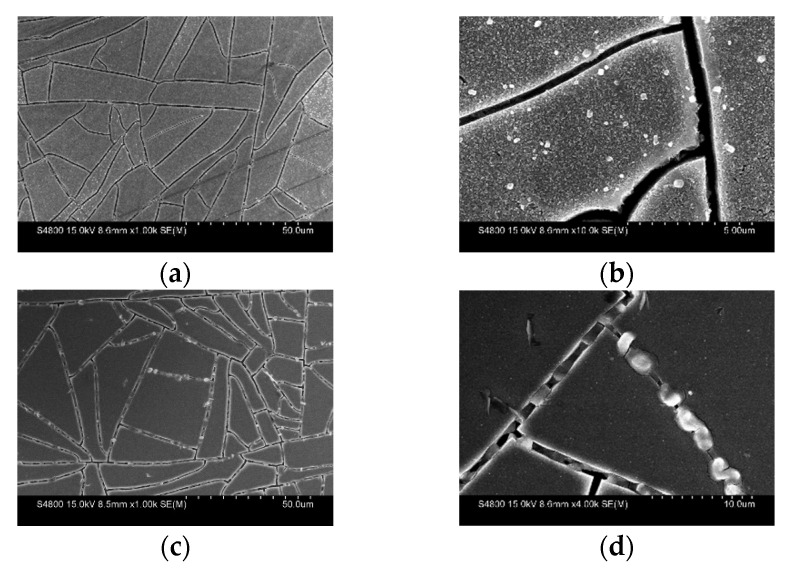
SEM observation of (**a**) MI-400 with the scale bar at 50 μm; (**b**) MI-400 with the scale bar at 5 μm; (**c**) MI-450 with the scale bar at 50 μm; (**d**) MI-450 with the scale bar at 10 μm.

At 400 °C, the surface morphology becomes nanoflaked with a surface area around 100 nm^2^. At the same time, the isolated bulk crystal around 500 nm in size is also observed on the surface, as shown in Figure 4a,b. When the temperature increases to 450 °C, the surface becomes bumpy. Isolated crystals and cracks can also be observed on the surface, as shown in Figure 4c,d.

These results illustrate that there are two reactions occurring during the sulfurization of MI@FTO, i.e., one starting at 250 °C, when the Mo_6_ clusters are sulfurized, leading to MoS_2_; one starting at 350 °C, not only when the Mo_6_ cluster sulfurization reaction occurs, but also when the Mo_6_ clusters catalyze the sulfurization of SnO_2_ into SnS.

### 3.2. Mechanism of the MI@FTO Sulfurized into MoS_2_ and SnS crystal Growth Hypothesis

**Figure 5:**
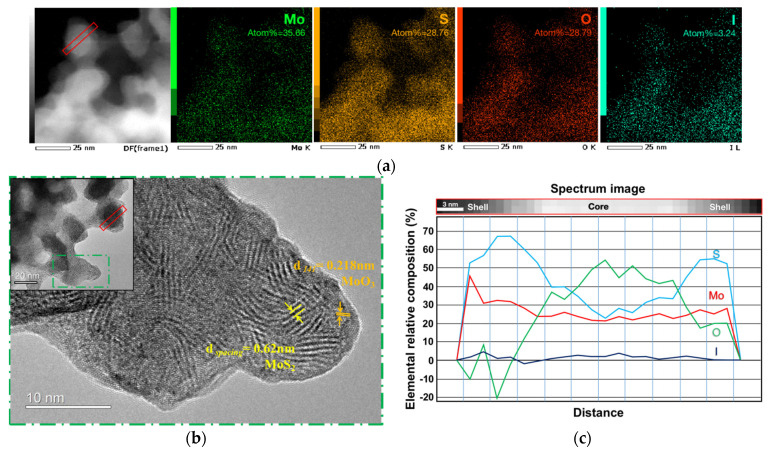
HRTEM observation of MI-250 powder scratched from thin films with the same observation area is marked in the figure. (**a**) Element mapping from HRTEM equipped with EDX device with the scale bar at 25 nm, and the elemental quantitative result is marked; (**b**) HRTEM image of MI-250; (**c**) EELS analysis of the MI-250 nanoparticles.

In order to clarify the sulfurization mechanism of the Mo_6_ cluster iodides, the MI-250 powder samples were scratched from the films for HRTEM observations (see Figure 5) in order to exclude interference from the FTO substrate and provide an easy sample preparation. 

It is noteworthy that the EDX analysis in Figure 5a demonstrates that the MI-250 contains Mo, S, O, and I elements, but not the Sn element, which means that SnO_2_ is not involved in the reaction below 250 ℃. The high-resolution analysis of the O element in the nanoparticle revealed that the Mo is oxidized. In addition to the MoS_2_ layer structure, the MoO_3_ crystal structure is also observed in Figure 5b. Moreover, the electron energy loss spectroscopy (EELS) analysis in Figure 5c revealed that there is a high concentration of Mo and S elements in the shell of the MI-250 nanoparticles but just a little oxygen. Conversely, the core contains a small amount of S and a high concentration of the Mo and O elements. This fact shows that, first, the film-bound water during the EPD process [45,58] serves as the O source, suggesting that the initial Mo_6_ cluster iodide is probably oxidized into MoO_3_. The H_2_S gas then serves as the S source for the subsequent sulfurization to produce MoS_2_ on the nanoparticle surface.

It has been reported that MoO_3_ can play the role of a precursor on the FTO substrates at low temperatures to be sulfurized into MoS_2_. On the other hand, SnO_2_ in the FTO coating is stable and does not participate in the reaction even when the temperature increases to 700 °C; thus, the final product, the MoS_2_ sulfurized from the MoO_3_ precursor, would not contain SnS. [59]. This fact confirms that the MoO_3_ used as a mid-product in this study is not the key material that catalyzes the SnO_2_:F layer on the FTO substrate to generate SnS under the H_2_/H_2_S.

**Figure 6:**
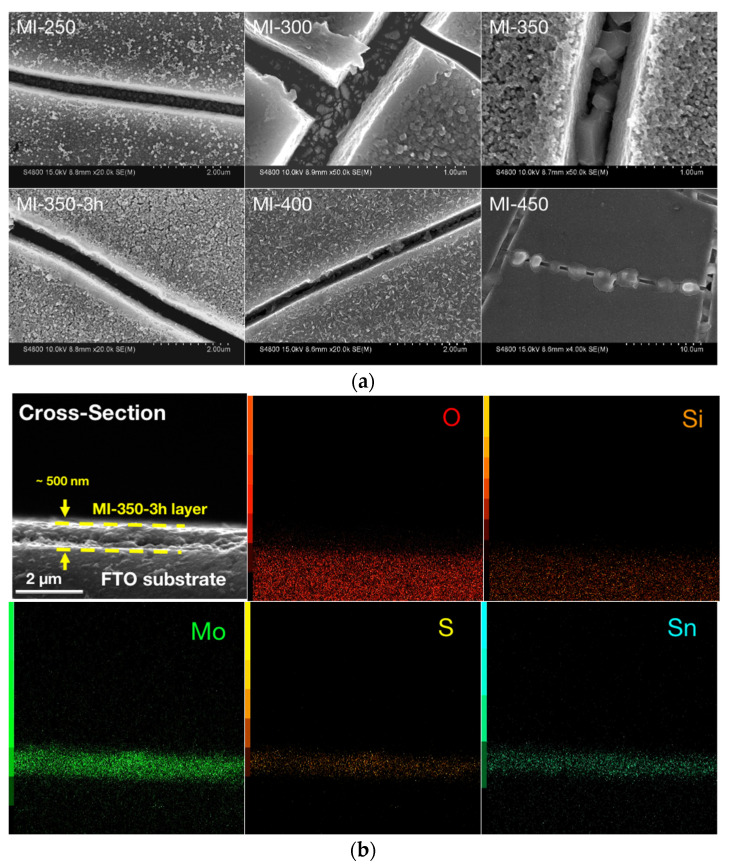
SEM observation of thin-film samples: (**a**) the cracks and (**b**) the cross-section of MI-350-3h.

Our study is the first report showing that Mo_6_ cluster iodides deposited on FTO substrates can generate SnS at 350 °C without external Sn sources, indicating that the Mo_6_ cluster iodides have a catalytic role. Although the reaction mechanism is still unclear, we attempted to provide some explanations for the growth of the SnS crystals. Figure 6a shows the FE-SEM observation focusing on the crack of the thin-film samples at different temperatures. The main morphology starting from the Mo_6_ cluster iodide nanoparticles is consistent with the observation shown in Figure 3. At high temperatures, cracks inevitably occur in the MI@FTO films, and the appearance of the cracks exposes the SnO_2_ layer to the H_2_/H_2_S gas flow and concomitantly to the Mo_6_-cluster-iodide-based layer. Multiple SnS_x_ phases are observed by heating of the blank FTO, free of Mo_6_ cluster iodides, starting from 350 °C (see Figure S1). However, the SnS crystals grow well from the exposed SnO_2_ surface and are catalyzed by the Mo_6_ cluster iodides at MI-350, and MI-400 also shows the bulk SnS crystal around 500 nm.

Based on this result, we extended the sulfurization time to 3 h (i.e., MI-350-3h), and the morphology of MI-350-3h still retains the shape of the nanoparticles with a diameter of about 50 nm, but these particles are sintered together. Figure 6b shows a cross-section of the MI-350-3h film sample with the presence of Sn that highlights the migration of the Sn in the Mo layer and a very uniform doping. This indicates that the SnO_2_ layer reacts with the Mo_6_ cluster iodide along the layer, not only on the top surface.

**Figure 7:**
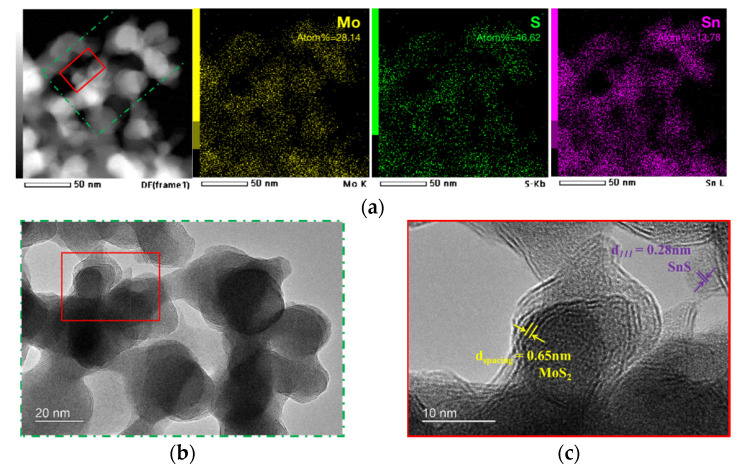
HRTEM observation of MI-350-3h powder scratched from thin film; the same observation area is marked in the figure. (**a**) Element mapping from HRTEM equipped with EDX device with the scale bar at 50 nm, and the elemental quantitative result is marked; (**b**) HRTEM image of MI-350-3h with the scale bar at 20 nm; (**c**) scale bar at 10 nm.

The MI-350-3h powder samples were also scratched from the films for the HRTEM observations (see Figure 7). The layer spacing of the 2D structure is 0.65 nm, which is consistent with MoS_2_. In addition, the lattice spacing of the nanocrystal structure is 0.28 nm, which is consistent with the (111) plane of SnS from the XRD results. The element mapping clearly shows the uniform distribution of the Mo, Sn, and S elements in the sample.

It is noteworthy that the EDX elemental quantitative analysis result shows that the content of the I element in MI-350-3h is I% = 0.07, while in the MI-250 sample, I% = 3.24. The content of the I element is much lower than those of the Mo and S elements in the sulfurized MI@FTO sample as the sulfur replaces the iodine ligand in the Mo_6_ cluster unit to form the Mo_x_S_y_ cluster. The Mo cluster structure gradually decomposes to form MoS_2_, which is the final product. The I_2_ gas generated by the decomposed Mo_x_ (1 ≤ x ≤ 6) cluster iodides reacts with H_2_/H_2_S and leaves the reaction chamber as a by-product in the gas flow. The Mo_6_ cluster iodides and I_2_ gas by-product during decomposition of the Mo_x_ cluster (1 ≤ x ≤ 6) may play an important role here to catalyze the SnO_2_:F layer to the SnS crystal.

The generation of halogen gas by-products during the sulfurization of the Mo_6_ clusters has also been reported in our previous studies [47]. By using the chemical vapor transport (CVT) method, the Mo_6_Br_12_ and sulfur powder were mixed and sealed in a quartz vacuum tube for the sulfurization reaction; the Br_2_ gas and MoBr_3_ by-product are retained in the tube and affects the pressure of the vacuum tube reaction system, which affects the final product, MoS_2_, as a 3D-formed nanosheet or single crystal.

**Figure 8:**
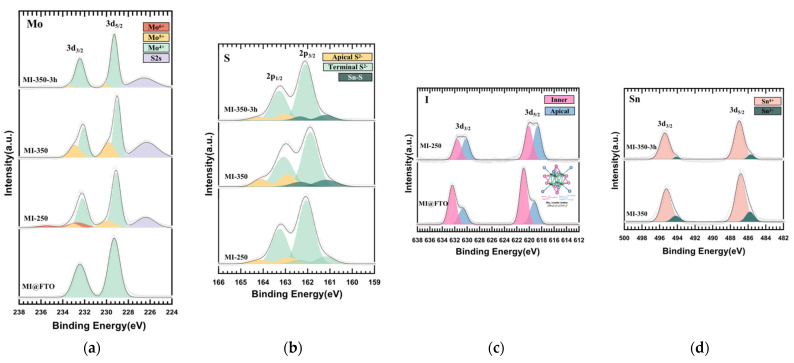
XPS result of MI@FTO, MI-250, MI-350, and MI-350-3h thin films: (**a**) Mo 3d spectrum; (**b**) S 2p spectrum; (**c**) I 3d spectrum; (**d**) Sn 3d spectrum.

Figure 8 shows the XPS results of four thin-film samples, i.e., MI@FTO, MI-250, MI-350, and MI-350-3h. One Mo 3d_5/2_-3d_3/2_ doublet peak is identified in the Mo 3d spectrum for the initial MI@FTO with binding energies of 229.3 eV–232.4 eV, suggesting the Mo^4+^ states. Moreover, two I 3d_5/2_-3d_3/2_ doublet peaks in the I 3d spectrum with a ratio of 2:1 are observed, which is consistent with our earlier research and correspond to the ratio of the inner ligands to apical ligands in the [{Mo_6_I^i^_8_}I^a^_4_(H_2_O)^a^_2_] cluster molecular structure combining H_2_O molecules [45,60]. Three Mo 3d_5/2_-3d_3/2_ doublet peaks are attributed to the Mo^4+^, Mo^5+^, and Mo^6+^ states for MI-250. According to the HRTEM result of MI-250, the Mo^4+^ states can be attributed to the MoS_2_ layered structure, whereas the Mo^5+^ states correspond to the Mo_x_S_y_ clusters [61,62,63,64]. Finally, the Mo^6+^ doublet peak must be due to MoO_x_, which is produced from the MI@FTO and bound water. Two doublet peaks are observed in the MI-250 I 3d spectrum with a ratio of roughly 1:1. The presence of the I 3d peak is not only consistent with the EDX elemental quantitative analysis result of the MI-250 sample (see Figure 5a), but the two doublet peaks representing the inner ligand and apical ligand also suggest that MI-250 contains a minor amount of molybdenum iodide sulfide clusters [65]. The three S 2p_3/2_-2p_1/2_ doublet peaks present in the MI-250 S 2p spectrum should all belong to the Mo-S compounds because MI-250 is devoid of Sn. The terminal S^2−^ in MoS_2_ is indicated by a major doublet at the binding energies of 162.1 eV–163.3 eV. On the other hand, a minor doublet is assigned to the apical/bridge S at the slightly higher binding energies of 162.9 eV–164.2 eV, which belong to the Mo_x_S_y_ phase with the Mo^5+^ state. An additional terminal S^2−^ doublet peak is also recognized as a minor doublet observed at the lower binding energies of 161.2 eV–162.4 eV. These low-binding energies are caused by the effect of H_2_ during the synthesis [66].

In the MI-350 and MI-350-3h samples, no Mo^6+^ state, representing MoO_x_, is found in the Mo 3d spectrum. The I 3d peak also disappears, proving that MoO_x_ as an intermediate product is completely sulfurized. The content of the Mo^5+^ ions in the product significantly decreases when the sulfurization time increases from 1 to 3 h, demonstrating that the Mo_x_S_y_ cluster phase gradually produces MoS_2_ that is compatible with the Raman results. 

Two Sn 3d 3d_5/2_-3d_3/2_ doublet peaks are observed in the Sn 3d spectrum of MI-350 and MI-350-3h, suggesting the Sn^4+^ and Sn^2+^ states. Sn^4+^ is associated with the Sn-doping into the MoS_2_ complex and the SnO_2_ substrate combined with the HRTEM results obtained for MI-350-3h, whereas the Sn^2+^ state corresponds to the SnS crystals. In the S 2p spectrum, the doublet peak corresponds to the Sn-S bonds. These results demonstrate that the SnO_2_-coated layer in the FTO substrate begins to take part in the reaction and synthesizes SnS, which is consistent with the XRD results, in which the Mo_6_ clusters demonstrate their outstanding catalytic capabilities.

Based on this result, the sulfurization reaction of the Mo_6_ cluster iodide thin films can be described as follows: first, the Mo_6_ clusters acquire bound water during the EPD coating, and the bound water reacts with the Mo_6_ cluster iodides at 250 °C to form MoO_x_; subsequently, MoO_x_ is sulfurized into MoS_2_ by H_2_S. In this process, the iodide ligand is gradually replaced by sulfur ligands and the Mo_6_ clusters are gradually decomposed. When the reaction temperature becomes higher or the reaction time becomes longer, the final product is the layered MoS_2_. Thus, the composition of the MI-250 thin film should be the MoO_x_-Mo_x_S_y_ cluster-MoS_2_ composite with doped iodide.

On the other hand, the main component of MI-300 should be a metal sulfide (oxysulfide) complex of Mo and Sn, while the components of MI-350 and MI-350-3h should be SnS-MoS_2_ nanoparticles. The MI-350-3h thin film has a porous sintered particles morphology. The distribution of the Mo and Sn elements is, thus, uniform, which is obtained at such a low synthesis temperature. Although the reaction mechanism of SnO_2_ catalyzed by Mo_6_ cluster iodides to form SnS is not known, it is worth further research.

### 3.3. NH_3_ Sensor Application

Ligated Mo_6_-cluster-containing compounds used for sensing activity are currently limited to oxygen sensor [67,68], pH sensing [69], biological sensing [70], and photo-response [58,71]. According to theoretical calculations, such compounds are predicted to have a good selectivity for NH_3_ in the NRR [29]. Previous studies have also demonstrated that they exhibit a photo-response and can react with NH_3_ to form Mo_x_N_y_ species at 400 °C [46,58,71]. Thus, we inferred that Mo_6_ cluster iodides should have a specific adsorption of NH_3_ even at room temperature and that this adsorption could cause a potential change when a voltage is applied to the MI@FTO, which was proved to be an ambipolar material (*E*_g_ = 1.9 eV). In addition, the nanoparticle morphology of the material could also increase the specific surface area and, thus, enhance the adsorption effect. Based on these facts, a simple MI@FTO sensor was designed and fabricated to verify this hypothesis (see Figure 1b).

The MI@FTO and sulfurized sample gas sensors were carefully tested at room temperature, 1 atmosphere, and in shaded light in order to investigate the sensing performance of NH_3_ under a N_2_ atmosphere for its potential applications in gas detection. The flow rate of the N_2_ gas stream was constant at 100 sccm. The flow rate of the NH_3_ gas was adjusted to achieve a change in the concentration of NH_3_ in the atmosphere. Its real-time current change was detected with the voltage of 0.1 V by flowing NH_3_ gas in the alumina tube. The gas sensing response (%) was calculated using the following equation:

(1) $$Response(\%)=\frac{{|I}_{g}-I_{0}|}{I_{0}}\times 100\%=\frac{∆I}{I_{0}}\times 100\%$$

where I_0_ is the base current, and I_g_ refers to the corresponding current with the NH_3_ gas *exposure.*

**Figure 9:**
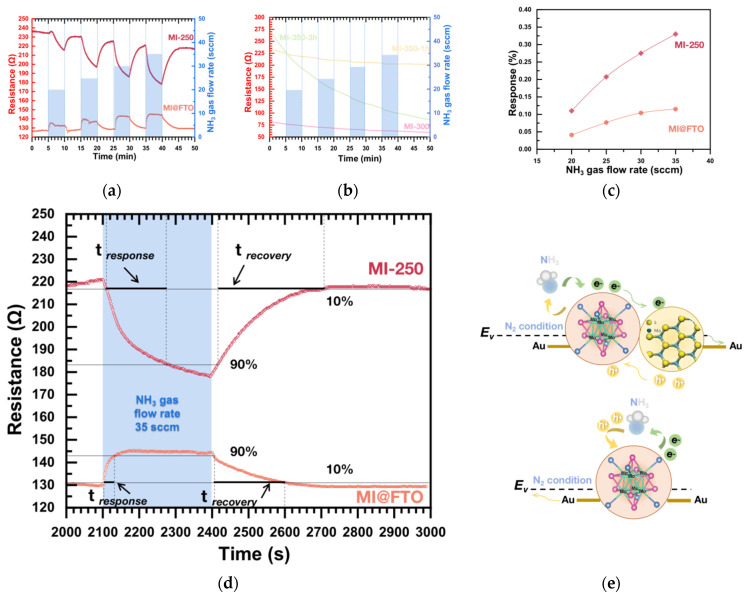
NH_3_ sensing performance of (**a**) MI@FTO, MI-250; (**b**) MI-300, MI-350, and MI-350-3h thin films; (**c**) calculated response of the MI@FTO and MI-250 gas sensor corresponding to gas flow rate of NH3; (**d**) transient response and recovery times of NH_3_ (35 sccm) for MI@FTO and MI-250 gas sensor; (**e**) schematic illustration of MI-250 gas sensor (**top**) and MI@FTO gas sensor (**bottom**) for NH_3_ under N_2_ condition.

Figure 9a shows the result of the NH_3_ sensing performance of the initial MI@FTO and MI-250 samples. The initial MI@FTO with the pure Mo_6_ cluster iodide film was, for the first time, used as a gas sensor and demonstrated a significant responsiveness. Gas sensing mechanisms can be interpreted as electron (or hole) transport between a sensing film and the target gas via charge transfer. Depending on whether the gas is oxidizing or reducing (e.g., NH_3_) and the sensing film is a p- or n-type semiconductor, electrons are withdrawn from or donated to the sensing film, which would cause the resistance of the sensing material to increase or decrease [72,73,74]. The initial MI@FTO shows an increased resistance when exposed to NH_3_ gas, while MI-250 shows a decrease in resistance, proving that MI@FTO shows the behavior of a p-type semiconductor, while MI-250 is an n-type conductor (see Figure 9e). This conversion could be the result of a sulfur defect and iodine doping [75,76,77].

The other samples (e.g., MI-300, MI-350-1h, and MI-350-3h) that contain Sn also exhibit the n-type characteristics but with a slight (or no) responsiveness to NH_3_ (see Figure 9b), even poorer than the MI@FTO, which may be due to the decomposition of the Mo cluster, thus reducing the activity site. The XRD results show that the composition of MI-300 is an extreme complex of Mo-Sn metal oxysulfur compounds, and the gas detector of MI-300 does not exhibit any selectivity for NH_3_ gas. For the MI-350-1h and MI-350-3h samples, XRD results show that the main component of the product is MoS_2_, which indicates that the Mo_6_ cluster structure has been largely decomposed and the SnS nanocrystal has been generated together, while the macrostructure of the material changes into a porous sinter. Both MI-350-1h and MI-350-3h show a poorer selectivity for NH_3_ compared to the initial MI@FTO, which may be mainly due to the decomposition of the Mo_6_ cluster. During the gas sensor measurements, most of the samples, including MI-350-3h, very quickly stabilize their resistance in ambient N_2_, while MI-350-1h continues to reduce its resistance in the ambient N_2_, proving, on the one hand, that it is also an n-type semiconductor, and on the other hand, it has a high capacity for adsorption than selectivity. This result is consistent with the XPS Mo 3d spectrum, which shows that MI-350-1h has more Mo^5+^ components compared to MI-350-3h, meaning that it has more active sites.

It is worth noting that when the sulfurization temperature is above 400 °C, the SnO_2_ layer in the FTO substrate will be destroyed and mixed into the sample (the HRTEM image of MI-400 is shown in Figure S2). Thus, the samples, which were sulfurized beyond 400 °C, were not made into a gas sensor for measurements.

Figure 9c shows the responses of MI@FTO and MI-250 based on different NH_3_ gas flow rates. As the measurement circuit is at a constant voltage (0.1 V), the response calculated for the current is actually equivalent to the rate of change of the sample’s resistance. The response is taken as an absolute value for the comparison; MI-250’s response rate improves 3 times versus that of the MI@FTO (11% to 31%) at an NH_3_ rate of 35 sccm. The increase in the response rate should be attributed to the activity sites provided by the Mo cluster [29] and the core–shell structure of MoO_3_-MoS_2_ [78], and probably attributed to the p–n heterojunction structure [79] between the Mo_6_ cluster and MoS_2_.

Based on the XRD results, the components of MI-250 can be described as a p-type Mo_x_ cluster (x ≤ 6) and sulfurized n-type MoS_2_ (and also MoO_x_). The XPS I 3d spectrum of MI-250 also shows two clear doublets, proving that the Mo_6_ cluster core is still present. Recently, it has been reported that when the Mo_6_ cluster iodide is immobilized on p-type graphene (Mo_6_@Graphene), this nanohybrid material exhibits a sensitivity to NO_2_ gas but not NH_3_ gas when made into the chemiresistive gas sensor. The response of Mo_6_@Graphene to NH_3_ is even lower than the response of the initial graphene to NH_3_, because the NH_3_ cannot exchange charges, and only few positive charges (hole) are transferred from the p-type graphene layer toward the Mo_6_ cluster [80]. These results demonstrate that the sulfurized strategy is effective in enhancing the NH_3_ gas response of the Mo_6_ cluster iodide, probably due to the p–n heterojunction structure between the Mo_6_ cluster and MoS_2_.

After the sulfurization process, the resistivity of the samples shows a good linear relationship at different temperatures (see Figure S3). It means that the sulfurized Mo_6_ cluster layer has good ohmic contact with the FTO electrode. Based on that, the resistance change of the gas sensor while it is exposed to the target gas can be reflected due to the negligible contact resistance at the interface. It is worth pointing out that the resistance of MI-250 and MI-300 varies very little with temperature (a small increase with temperature), which indicates that MI-250 has good stability as a gas sensor. In contrast, the resistance of MI-350, MI-350-3h, and MI-400 decreases rapidly as temperature rises, indicating that they have a high potential for future thermosensitive applications.

Figure 9d, based on Figure 9a, shows the response time and recovery time of MI@FTO and MI-250 at the NH_3_ gas flow rate of 35 sccm. The response time was measured from the resistance point of the sample increasing by 10% to 90% of an end point (the maximum resistance point), while the sample was exposed to the target gas, and the recovery time was measured at the point where the resistance decreased from 90% of an end point to the 10% resistance point after the target gas was turned off. The response and recovery times for the NH_3_ detection at 35 sccm by the MI@FTO are 29 s and 198 s, respectively. In comparison, the MI-250 response and recovery times are 174 s and 254 s, respectively, both significantly lower than the performance of the MI@FTO. MI@FTO’s fast response and recovery are attributed to its Mo_6_ cluster core [29].

After the gas sensor measurement, the final resistance of all the samples in this 45-min long test does not go back to zero when the NH_3_ gas turns off, which may be due to the fact that the sensor surface is not fully cleaned and some NH_3_ is still adsorbed. This behavior may be related to operating temperature and gas flow rate.

The sulfurization process starting from the Mo_6_ cluster iodides as the precursor leading to the expected MoS_2_ product and their NH_3_ sensing enhancement is evidence that this novel synthesis strategy is feasible. The transition of the Mo_6_-cluster-iodide-based films from p-type to n-type semiconductors due to sulfurization reactions also opens up new design possibilities for future device applications, such as p–n junctions.

## 4. Conclusions

This study established a simple and efficient method for the synthesis of Mo cluster-MoS_2_ nanocomposites, which not only demonstrates the feasibility of sulfuring Mo_6_ cluster iodides as precursors to generate MoS_2_, but also directly allows the synthesis of nanocomposite thin films in just one step. This study also explored the performance of Mo_6_ cluster iodides and Mo cluster-MoS_2_ nanocomposites as gas sensors for the first time. It was proved that the Mo_6_ cluster iodide gas sensor for NH_3_ at room temperature responds as a p-type semiconductor. After the sulfurization process at low temperature (250 °C), the response to NH_3_ gas increased by three times while the conversion from the p-type semiconductor to n-type occurred. Our results obtained in this study show the possibilities of Mo_6_ clusters and Mo-cluster-based MoS_2_ material for future applications.

## Data Availability

All data concerning this study are contained in the present manuscript, in previous articles or Supplementary Materials, whose references have been provided.

## Supplementary Materials

The following supporting information can be downloaded at: https://www.mdpi.com/article/10.3390/nano13030478/s1, Figure S1: GIXRD pattern of FTO substrate sulfurized at different temperatures; Figure S2: HRTEM observation of MI-400 powder scratched from thin film; the same observation area is marked in the figure (**a**) HRTEM image of MI-400 with the scale bar at 100 nm; (**b**) scale bar at 10 nm; (**c**) Element mapping from HRTEM equipped with EDX device with the scale bar at 25 nm; Figure S3: Electrical measurements of the sulfurized samples at different temperatures, with a blank FTO substrate inserted at the top right as a reference.

## References

[1] Fiori G., Bonaccorso F., Iannaccone G., Palacios T., Neumaier D., Seabaugh A., Banerjee S.K., Colombo L. (2014). Electronics Based on Two-Dimensional Materials. Nat. Nanotechnol..

[2] Zeng H., Dai J., Yao W., Xiao D., Cui X. (2012). Valley Polarization in MoS_2_ Monolayers by Optical Pumping. Nat. Nanotechnol..

[3] Mak K.F., Lee C., Hone J., Shan J., Heinz T.F. (2010). Atomically Thin MoS_2_: A New Direct-Gap Semiconductor. Phys. Rev. Lett..

[4] Chen W., Santos E.J.G., Zhu W., Kaxiras E., Zhang Z. (2013). Tuning the Electronic and Chemical Properties of Monolayer MoS_2_ Adsorbed on Transition Metal Substrates. Nano Lett..

[5] Chen M., Nam H., Wi S., Priessnitz G., Gunawan I.M., Liang X. (2014). Multibit Data Storage States Formed in Plasma-Treated MoS_2_ Transistors. ACS Nano.

[6] Kang J., Liu W., Banerjee K. (2014). High-Performance MoS_2_ Transistors with Low-Resistance Molybdenum Contacts. Appl. Phys. Lett..

[7] Tsai M.-L., Su S.-H., Chang J.-K., Tsai D.-S., Chen C.-H., Wu C.-I., Li L.-J., Chen L.-J., He J.-H. (2014). Monolayer MoS_2_ Heterojunction Solar Cells. ACS Nano.

[8] Zhao Y., Ouyang G. (2019). Thickness-Dependent Photoelectric Properties of MoS_2_/Si Heterostructure Solar Cells. Sci. Rep..

[9] Mao J., Wang Y., Zheng Z., Deng D. (2018). The Rise of Two-Dimensional MoS_2_ for Catalysis. Front. Phys..

[10] Li H., Tsai C., Koh A.L., Cai L., Contryman A.W., Fragapane A.H., Zhao J., Han H.S., Manoharan H.C., Abild-Pedersen F. (2015). Activating and Optimizing MoS_2_ Basal Planes for Hydrogen Evolution through the Formation of Strained Sulphur Vacancies. Nat. Mater..

[11] Cao Y. (2021). Roadmap and Direction toward High-Performance MoS_2_ Hydrogen Evolution Catalysts. ACS Nano.

[12] Akbari E., Jahanbin K., Afroozeh A., Yupapin P., Buntat Z. (2018). Brief Review of Monolayer Molybdenum Disulfide Application in Gas Sensor. Phys. B Condens. Matter.

[13] Liu B., Chen L., Liu G., Abbas A.N., Fathi M., Zhou C. (2014). High-Performance Chemical Sensing Using Schottky-Contacted Chemical Vapor Deposition Grown Monolayer MoS_2_ Transistors. ACS Nano.

[14] Donarelli M., Ottaviano L. (2018). 2D Materials for Gas Sensing Applications: A Review on Graphene Oxide, MoS_2_, WS_2_ and Phosphorene. Sensors.

[15] Kibsgaard J., Chen Z., Reinecke B.N., Jaramillo T.F. (2012). Engineering the Surface Structure of MoS_2_ to Preferentially Expose Active Edge Sites for Electrocatalysis. Nat. Mater..

[16] Li X., Li T., Ma Y., Wei Q., Qiu W., Guo H., Shi X., Zhang P., Asiri A.M., Chen L. (2018). Boosted Electrocatalytic N_2_ Reduction to NH_3_ by Defect-Rich MoS_2_ Nanoflower. Adv. Energy Mater..

[17] Le D., Rawal T.B., Rahman T.S. (2014). Single-Layer MoS_2_ with Sulfur Vacancies: Structure and Catalytic Application. J. Phys. Chem. C.

[18] Li L., Qin Z., Ries L., Hong S., Michel T., Yang J., Salameh C., Bechelany M., Miele P., Kaplan D. (2019). Role of Sulfur Vacancies and Undercoordinated Mo Regions in MoS_2_ Nanosheets toward the Evolution of Hydrogen. ACS Nano.

[19] Fei H., Guo T., Xin Y., Wang L., Liu R., Wang D., Liu F., Wu Z. (2022). Sulfur Vacancy Engineering of MoS_2_ via Phosphorus Incorporation for Improved Electrocatalytic N_2_ Reduction to NH_3_. Appl. Catal. B Environ..

[20] Benck J.D., Hellstern T.R., Kibsgaard J., Chakthranont P., Jaramillo T.F. (2014). Catalyzing the Hydrogen Evolution Reaction (HER) with Molybdenum Sulfide Nanomaterials. ACS Catal..

[21] Tsai C., Li H., Park S., Park J., Han H.S., Nørskov J.K., Zheng X., Abild-Pedersen F. (2017). Electrochemical Generation of Sulfur Vacancies in the Basal Plane of MoS_2_ for Hydrogen Evolution. Nat. Commun..

[22] Kumar R., Zheng W., Liu X., Zhang J., Kumar M. (2020). MoS_2_-Based Nanomaterials for Room-Temperature Gas Sensors. Adv. Mater. Technol..

[23] Ramanathan A.A. (2018). Defect Functionalization of MoS_2_ Nanostructures as Toxic Gas Sensors: A Review. IOP Conf. Ser. Mater. Sci. Eng..

[24] Burman D., Ghosh R., Santra S., Ray S.K., Guha P.K. (2017). Role of Vacancy Sites and UV-Ozone Treatment on Few Layered MoS_2_ nanoflakes for Toxic Gas Detection. Nanotechnology.

[25] Xie J., Zhang H., Li S., Wang R., Sun X., Zhou M., Zhou J., Lou X.W.D., Xie Y. (2013). Defect-Rich MoS_2_ Ultrathin Nanosheets with Additional Active Edge Sites for Enhanced Electrocatalytic Hydrogen Evolution. Adv. Mater..

[26] Zhang Z., Dong Y., Sun H., Liu G., Liu S., Yang X. (2019). Defect-Rich 2D Reticulated MoS_2_ Monolayers: Facile Hydrothermal Preparation and Marvellous Photoelectric Properties. J. Taiwan Inst. Chem. Eng..

[27] Syari’ati A., Kumar S., Zahid A., El Yumin A.A., Ye J., Rudolf P. (2019). Photoemission Spectroscopy Study of Structural Defects in Molybdenum Disulfide (MoS_2_) Grown by Chemical Vapor Deposition (CVD). Chem. Commun..

[28] Young B.T., Pathan M.A.K., Jiang T., Le D., Marrow N., Nguyen T., Jordan C.E., Rahman T.S., Popolan-Vaida D.M., Vaida M.E. (2020). Catalytic C_2_H_2_ Synthesis via Low Temperature CO Hydrogenation on Defect-Rich 2D-MoS_2_ and 2D-MoS_2_ Decorated with Mo Clusters. J. Chem. Phys..

[29] Zhang Z., Xu X. (2022). Mechanistic Study on Enhanced Electrocatalytic Nitrogen Reduction Reaction by Mo Single Clusters Supported on MoS_2_. ACS Appl. Mater. Interfaces.

[30] Huang W., Wang X., Ji X., Zhang Z., Jin C. (2018). In-Situ Fabrication of Mo_6_S_6_-Nanowire-Terminated Edges in Monolayer Molybdenum Disulfide. Nano Res..

[31] Wang X.-W., Hou L.-F., Huang W., Ren X.-B., Ji W., Jin C.-H. (2021). Mass Transport Induced Structural Evolution and Healing of Sulfur Vacancy Lines and Mo Chain in Monolayer MoS_2_. Rare Met..

[32] Viršek M., Novak N., Filipič C., Kump P., Remškar M., Kutnjak Z. (2012). Transport Properties in MoS_2_ Selective Morphology System. J. Appl. Phys..

[33] Viršek M., Krause M., Kolitsch A., Mrzel A., Iskra I., Škapin S.D., Remškar M. (2010). The Transformation Pathways of Mo_6_S_2_I_8_ Nanowires into Morphology-Selective MoS_2_ Nanostructures. J. Phys. Chem. C.

[34] Chevrel R., Sergent M., Prigent J. (1971). Sur de Nouvelles Phases Sulfurées Ternaires Du Molybdène. J. Solid State Chem..

[35] Perrin A., Perrin C. (2012). The Molybdenum and Rhenium Octahedral Cluster Chalcohalides in Solid State Chemistry: From Condensed to Discrete Cluster Units. Comptes Rendus Chim..

[36] Fedorov V. (2014). Metal Clusters. As They Were Born in Siberia. J. Clust. Sci..

[37] Vorotnikova N.A., Vorotnikov Y.A., Novozhilov I.N., Syrokvashin M.M., Nadolinny V.A., Kuratieva N.V., Benoit D.M., Mironov Y.V., Walton R.I., Clarkson G.J. (2017). 23-Electron Octahedral Molybdenum Cluster Complex [{Mo_6_I_8_}Cl_6_]^−^. Inorg. Chem..

[38] Nguyen N.T.K., Lebastard C., Wilmet M., Dumait N., Renaud A., Cordier S., Ohashi N., Uchikoshi T., Grasset F. (2022). A Review on Functional Nanoarchitectonics Nanocomposites Based on Octahedral Metal Atom Clusters (Nb_6_, Mo_6_, Ta_6_, W_6_, Re_6_): Inorganic 0D and 2D Powders and Films. Sci. Technol. Adv. Mater..

[39] Costuas K., Garreau A., Bulou A., Fontaine B., Cuny J., Gautier R., Mortier M., Molard Y., Duvail J.-L., Faulques E. (2015). Combined Theoretical and Time-Resolved Photoluminescence Investigations of [Mo_6_Br^i^_8_Br^a^_6_]^2−^ Metal Cluster Units: Evidence of Dual Emission. Phys. Chem. Chem. Phys..

[40] Sokolov M.N., Mihailov M.A., Peresypkina E.V., Brylev K.A., Kitamura N., Fedin V.P. (2011). Highly Luminescent Complexes [Mo_6_X_8_(N-C_3_F_7_COO)_6_]^2−^ (X = Br, I). Dalton Trans..

[41] Dierre B., Costuas K., Dumait N., Paofai S., Amela-Cortes M., Molard Y., Grasset F., Cho Y., Takahashi K., Ohashi N. (2017). Mo_6_ Cluster-Based Compounds for Energy Conversion Applications: Comparative Study of Photoluminescence and Cathodoluminescence. Sci. Technol. Adv. Mater..

[42] Dybtsev D., Serre C., Schmitz B., Panella B., Hirscher M., Latroche M., Llewellyn P.L., Cordier S., Molard Y., Haouas M. (2010). Influence of [Mo_6_Br_8_F_6_]^2−^ Cluster Unit Inclusion within the Mesoporous Solid MIL-101 on Hydrogen Storage Performance. Langmuir.

[43] Barras A., Das M.R., Devarapalli R.R., Shelke M.V., Cordier S., Szunerits S., Boukherroub R. (2013). One-Pot Synthesis of Gold Nanoparticle/Molybdenum Cluster/Graphene Oxide Nanocomposite and Its Photocatalytic Activity. Appl. Catal. B Environ..

[44] Nguyen N.T.K., Renaud A., Dierre B., Bouteille B., Wilmet M., Dubernet M., Ohashi N., Grasset F., Uchikoshi T. (2018). Extended Study on Electrophoretic Deposition Process of Inorganic Octahedral Metal Clusters: Advanced Multifunctional Transparent Nanocomposite Thin Films. Bull. Chem. Soc. Jpn..

[45] Renaud A., Jouan P.-Y., Dumait N., Ababou-Girard S., Barreau N., Uchikoshi T., Grasset F., Jobic S., Cordier S. (2021). Evidence of the Ambipolar Behavior of Mo_6_ Cluster Iodides in All-Inorganic Solar Cells: A New Example of Nanoarchitectonic Concept. ACS Appl. Mater. Interfaces.

[46] Guy K., Tessier F., Kaper H., Grasset F., Dumait N., Demange V., Nishio M., Matsushita Y., Matsui Y., Takei T. (2020). Original Synthesis of Molybdenum Nitrides Using Metal Cluster Compounds as Precursors: Applications in Heterogeneous Catalysis. Chem. Mater..

[47] Zhang M.Q., Grasset F., Dumait N., Cordier S., Shimada T., Uchikoshi T. (2021). Effect of Sulfurization Process on Octahedral Molybdenum Cluster from Mo_6_ Cluster to MoS_2_ Nanosheet. Key Eng. Mater..

[48] Saito G., Hosoda H., Yoshida Y., Hagiwara J., Nishimura K., Yamochi H., Otsuka A., Hiramatsu T., Shimazaki Y., Kirakci K. (2012). Synthesis and Properties of Charge-Transfer Solids with Cluster Units [Mo_6_X_14_]^2−^ (X = Br, I). J. Mater. Chem..

[49] Kirakci K., Cordier S., Perrin C. (2005). Synthesis and Characterization of Cs_2_Mo_6_X_14_ (X = Br or I) Hexamolybdenum Cluster Halides: Efficient Mo_6_ Cluster Precursors for Solution Chemistry Syntheses. Z. Anorg. Und Allg. Chem..

[50] Nguyen T.K.N., Grasset F., Dierre B., Matsunaga C., Cordier S., Lemoine P., Ohashi N., Uchikoshi T. (2016). Fabrication of Transparent Thin Film of Octahedral Molybdenum Metal Clusters by Electrophoretic Deposition. ECS J. Solid State Sci. Technol..

[51] Renaud A., Nguyen T.K.N., Grasset F., Raissi M., Guillon V., Delabrouille F., Dumait N., Jouan P.-Y., Cario L., Jobic S. (2019). Preparation by Electrophoretic Deposition of Molybdenum Iodide Cluster-Based Functional Nanostructured Photoelectrodes for Solar Cells. Electrochim. Acta.

[52] Akram H., Mateos-Pedrero C., Gallegos-Suarez E., Chafik T., Guerrero-Ruiz A., Rodríguez-Ramos I. (2017). Effect of Surfactant Concentration on the Morphology of Mo_x_S_y_ Nanoparticles Prepared by a Solvothermal Route. Green Process. Synth..

[53] Polivtseva S., Acik I.O., Katerski A., Mere A., Mikli V., Krunks M. (2014). Spray Pyrolysis Deposition of Sn_x_S_y_ Thin Films. Energy Procedia.

[54] Kaidi Z., Boulanger C., Lecuire J.M., Lemée N., Guilloux-Viry M., Perrin A. (1999). Ternary Molybdenum Cluster Sulfides: Electrochemical and Chemical Behavior of in Situ Pulsed Laser Deposited Thin Films. Solid State Sci..

[55] Genuit D., Bezverkhyy I., Afanasiev P. (2005). Solution Preparation of the Amorphous Molybdenum Oxysulfide MoOS_2_ and Its Use for Catalysis. J. Solid State Chem..

[56] Liu G., Li Z., Hasan T., Chen X., Zheng W., Feng W., Jia D., Zhou Y., Hu P. (2017). Vertically Aligned Two-Dimensional SnS_2_ Nanosheets with a Strong Photon Capturing Capability for Efficient Photoelectrochemical Water Splitting. J. Mater. Chem. A.

[57] Burton L.A., Colombara D., Abellon R.D., Grozema F.C., Peter L.M., Savenije T.J., Dennler G., Walsh A. (2013). Synthesis, Characterization, and Electronic Structure of Single-Crystal SnS, Sn_2_S_3_, and SnS_2_. Chem. Mater..

[58] Harada K., Nguyen T.K.N., Grasset F., Comby-Zerbino C., MacAleese L., Chirot F., Dugourd P., Dumait N., Cordier S., Ohashi N. (2022). Light-Dependent Ionic-Electronic Conduction in an Amorphous Octahedral Molybdenum Cluster Thin Film. NPG Asia Mater..

[59] Chen Z., Cummins D., Reinecke B.N., Clark E., Sunkara M.K., Jaramillo T.F. (2011). Core–Shell MoO_3_–MoS_2_ Nanowires for Hydrogen Evolution: A Functional Design for Electrocatalytic Materials. Nano Lett..

[60] Saito N., Cordier S., Lemoine P., Ohsawa T., Wada Y., Grasset F., Cross J.S., Ohashi N. (2017). Lattice and Valence Electronic Structures of Crystalline Octahedral Molybdenum Halide Clusters-Based Compounds, Cs_2_[Mo_6_X_14_] (X = Cl, Br, I), Studied by Density Functional Theory Calculations. Inorg. Chem..

[61] Rodenes M., Gonell F., Martín S., Corma A., Sorribes I. (2022). Molecularly Engineering Defective Basal Planes in Molybdenum Sulfide for the Direct Synthesis of Benzimidazoles by Reductive Coupling of Dinitroarenes with Aldehydes. JACS Au.

[62] Fominski V., Demin M., Nevolin V., Fominski D., Romanov R., Gritskevich M., Smirnov N. (2020). Reactive Pulsed Laser Deposition of Clustered-Type MoS_x_ (X ~ 2, 3, and 4) Films and Their Solid Lubricant Properties at Low Temperature. Nanomaterials.

[63] Lee C.-H., Lee S., Lee Y.-K., Jung Y.C., Ko Y.-I., Lee D.C., Joh H.-I. (2018). Understanding the Origin of Formation and Active Sites for Thiomolybdate [Mo_3_S_13_]^2–^ Clusters as Hydrogen Evolution Catalyst through the Selective Control of Sulfur Atoms. ACS Catal..

[64] Seo B., Jung G.Y., Lee S.J., Baek D.S., Sa Y.J., Ban H.W., Son J.S., Park K., Kwak S.K., Joo S.H. (2019). Monomeric MoS_4_^2–^-Derived Polymeric Chains with Active Molecular Units for Efficient Hydrogen Evolution Reaction. ACS Catal..

[65] Pritzi M., Pascher T.F., Grutza M.-L., Kurz P., Ončák M., Beyer M.K. (2022). Decomposition of Halogenated Molybdenum Sulfide Dianions [Mo_3_S_7_X_6_]^2–^ (X = Cl, Br, I). J. Am. Soc. Mass Spectrom..

[66] de Jong A.M., Borg H.J., van IJzendoorn L.J., Soudant V.G.F.M., de Beer V.H.J., van Veen J.A.R., Niemantsverdriet J.W. (1993). Sulfidation Mechanism by Molybdenum Catalysts Supported on Silica/Silicon(100) Model Support Studied by Surface Spectroscopy. J. Phys. Chem..

[67] Ghosh R.N., Baker G.L., Ruud C., Nocera D.G. (1999). Fiber-Optic Oxygen Sensor Using Molybdenum Chloride Cluster Luminescence. Appl. Phys. Lett..

[68] Ghosh R.N., Askeland P.A., Kramer S., Loloee R. (2011). Optical Dissolved Oxygen Sensor Utilizing Molybdenum Chloride Cluster Phosphorescence. Appl. Phys. Lett..

[69] Khazieva A., Kholin K., Nizameev I., Brylev K., Kashnik I., Voloshina A., Lyubina A., Gubaidullin A., Daminova A., Petrov K. (2021). Surface Modification of Silica Nanoparticles by Hexarhenium Anionic Cluster Complexes for PH-Sensing and Staining of Cell Nuclei. J. Colloid Interface Sci..

[70] Elistratova J., Mikhailov M., Burilov V., Babaev V., Rizvanov I., Mustafina A., Abramov P., Sokolov M., Konovalov A., Fedin V. (2014). Supramolecular Assemblies of Triblock Copolymers with Hexanuclear Molybdenum Clusters for Sensing Antibiotics in Aqueous Solutions via Energy Transfer. RSC Adv..

[71] Nguyen T.K.N., Dumait N., Grasset F., Cordier S., Berthebaud D., Matsui Y., Ohashi N., Uchikoshi T. (2020). Zn–al Layered Double Hydroxide Film Functionalized by a Luminescent Octahedral Molybdenum Cluster: Ultraviolet–Visible Photoconductivity Response. ACS Appl. Mater. Interfaces.

[72] Cao J., Chen Q., Wang X., Zhang Q., Yu H.-D., Huang X., Huang W. (2021). Recent Development of Gas Sensing Platforms Based on 2D Atomic Crystals. Research.

[73] Late D.J., Huang Y.-K., Liu B., Acharya J., Shirodkar S.N., Luo J., Yan A., Charles D., Waghmare U.V., Dravid V.P. (2013). Sensing Behavior of Atomically Thin-Layered MoS_2_ Transistors. ACS Nano.

[74] Wang Y., Duan L., Deng Z., Liao J. (2020). Electrically Transduced Gas Sensors Based on Semiconducting Metal Oxide Nanowires. Sensors.

[75] Shang H., Wang T., Zhang W. (2019). Sulfur Vacancy Formation at Different MoS_2_ Edges during Hydrodesulfurization Process: A DFT Study. Chem. Eng. Sci..

[76] Yang L., Majumdar K., Liu H., Du Y., Wu H., Hatzistergos M., Hung P.Y., Tieckelmann R., Tsai W., Hobbs C. (2014). Chloride Molecular Doping Technique on 2D Materials: WS_2_ and MoS_2_. Nano Lett..

[77] Murugan P., Kumar V., Kawazoe Y., Ota N. (2007). Assembling Nanowires from Mo−S Clusters and Effects of Iodine Doping on Electronic Structure. Nano Lett..

[78] Yuan Z., Liu Y., Zhang J., Meng F., Zhang H. (2021). Rose-like MoO_3_/MoS_2_/RGO Low-Temperature Ammonia Sensors Based on Multigas Detection Methods. IEEE Trans. Instrum. Meas..

[79] Kim Y., Lee S., Song J., Ko K.Y., Woo W.J., Lee S.W., Park M., Lee H., Lee Z., Choi H. (2020). 2D Transition Metal Dichalcogenide Heterostructures for P- and N-Type Photovoltaic Self-Powered Gas Sensor. Adv. Funct. Mater..

[80] Casanova-Chafer J., Garcia-Aboal R., Atienzar P., Feliz M., Llobet E. (2022). Octahedral Molybdenum Iodide Clusters Supported on Graphene for Resistive and Optical Gas Sensing. ACS Appl. Mater. Interfaces.

