# Supplementary material for: Enhanced NH3 Sensing Performance of Mo Cluster-MoS2 Nanocomposite Thin Films via the Sulfurization of Mo6 Cluster Iodides Precursor
Source: Nanomaterials (Basel). 2023 Jan 24;13(3):478. doi: 10.3390/nano13030478 (PMC9921185; doi:10.3390/nano13030478)
Supplement: Supplementary file 1 [file nanomaterials-13-00478-s001.zip › nanomaterials-2099572-supplementary.pdf]

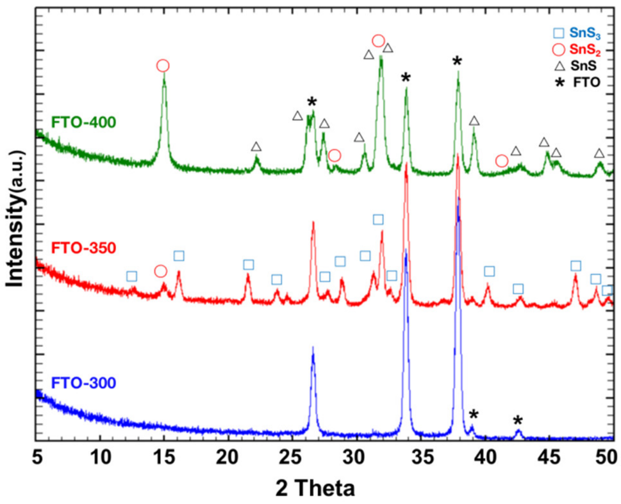

(a)

Figure S1. GIXRD pattern of FTO substrate sulfurized at different temperatures, w = 0.8.

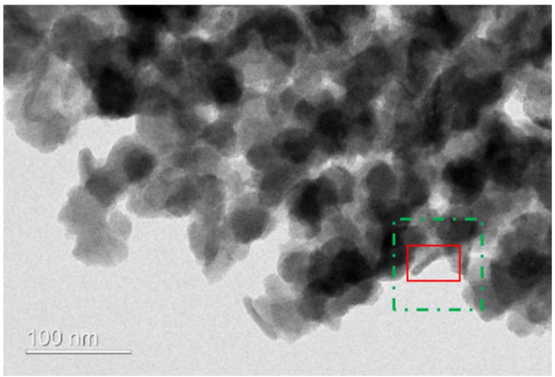

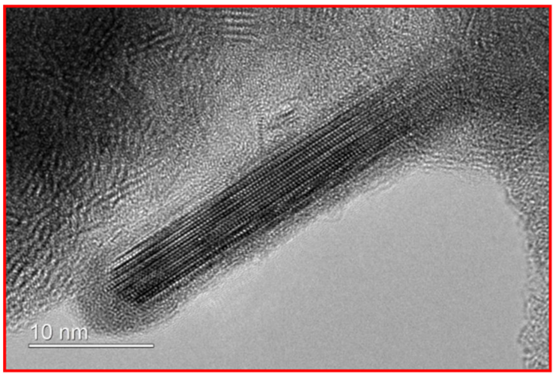

(a) **(b)**

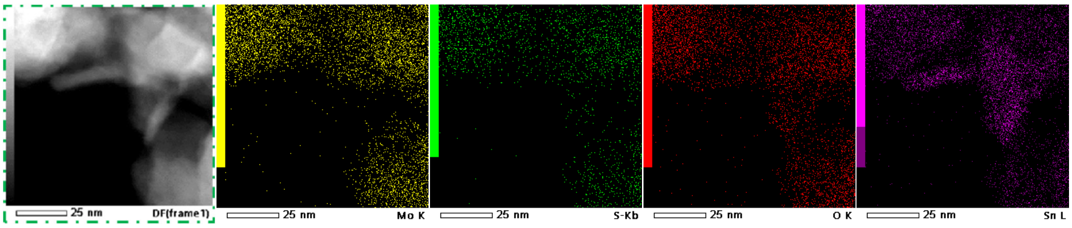

(c)

Figure S2. HRTEM observation of MI-400 powder scratched from thin film; the same observation area is marked in the figure (**a**) HRTEM image of MI-400 with the scale bar at 100 nm; (**b**) scale bar at 10 nm; (**c**) Element mapping from HRTEM equipped with EDX device with the scale bar at 25 nm.

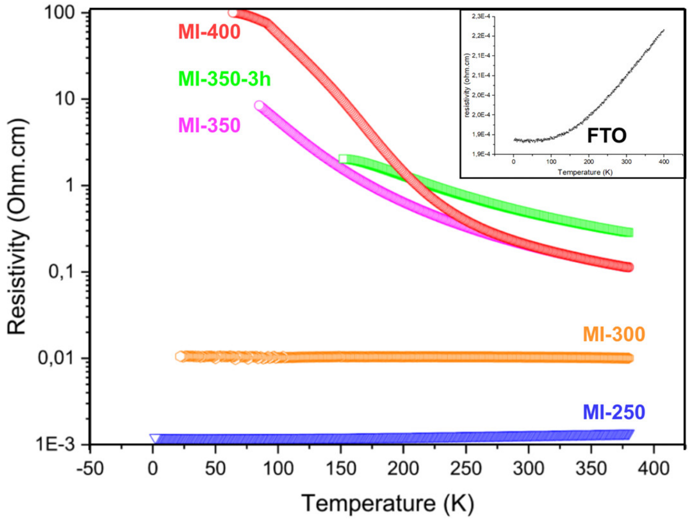

Figure S3. Electrical measurements of the sulfurized samples at different temperatures, with a blank FTO substrate inserted at the top right as a reference.
